# Silicon-induced reversibility of cadmium toxicity in rice

**DOI:** 10.1093/jxb/erw175

**Published:** 2016-04-27

**Authors:** Muhammad Ansar Farooq, Amelie Detterbeck, Stephan Clemens, Karl-Josef Dietz

**Affiliations:** ^1^Biochemistry and Physiology of Plants, Faculty of Biology, W5-134, Bielefeld University, University Street 25, D-33501 Bielefeld, Germany; ^2^Department of Plant Physiology, University of Bayreuth, University Street 30, D-95440 Bayreuth, Germany

**Keywords:** Ascorbate, Cd toxicity, glutathione, oxidative stress, photosynthesis, rice, SAP, silicon, transcript regulation.

## Abstract

Silicon (Si) modulates tolerance to abiotic stresses, but little is known about the reversibility of stress effects by supplementing previously stressed plants with Si. This is surprising since recovery experiments might allow mechanisms of Si-mediated amelioration to be addressed. Rice was exposed to 10 µM CdCl_2_ for 4 d in hydroponics, followed by 0.6mM Si(OH)_4_ supplementation for 4 d. Si reversed the effects of Cd, as reflected in plant growth, photosynthesis, elemental composition, and some biochemical parameters. Cd-dependent deregulation of nutrient homeostasis was partially reversed by Si supply. Photosynthetic recovery within 48h following Si supply, coupled with strong stimulation of the ascorbate–glutathione system, indicates efficient activation of defense. The response was further verified by transcript analyses with emphasis on genes encoding members of the stress-associated protein (SAP) family. The transcriptional response to Cd was mostly reversed following Si supply. Reprogramming of the Cd response was obvious for *Phytochelatin synthase 1*, *SAP1* , *SAP14*, and the transcription factor genes *AP2/Erf020*, *Hsf31*, and *NAC6* whose transcript levels were strongly activated in roots of Cd-stressed rice, but down-regulated in the presence of Si. These findings, together with changes in biochemical parameters, highlight the significance of Si in growth recovery of Cd-stressed rice and indicate a decisive role for readjusting cell redox homeostasis.

## Highlight

Rice exposed to a toxic cadmium concentration recovers from toxicity symptoms after supplementation of the medium with silicon, and transcript levels of marker genes are readjusted to that in unstressed conditions.

## Introduction

Cadmium (Cd) is almost ubiquitously present as an environmental pollutant in the rice-growing regions of the world and threatens the quality of harvested rice grains for human food (Zhu *et al.*, 2008; Williams *et al.*, 2009; Zhuang *et al.*, 2009). Excessive use of phosphate fertilizers and sewage sludge in irrigated rice areas are the major sources of Cd contamination. Cd accumulation in rice grains can cause severe human health problems especially in areas where rice is a dietary staple (Kikuchi *et al.*, 2007; Takahashi *et al.*, 2011). Recent risk assessments suggest that there is no safety margin between current dietary Cd exposure and potential development of adverse health effects (Clemens *et al.*, 2013).

Plants exposed to Cd encounter toxicity effects by interference with proteins and lipids and production of reactive oxygen species (ROS). Disturbances in the functional components of thylakoid membranes, integral for photosynthetic activity, are also considered as potential sites for metal-induced toxicity (Shah *et al.*, 2001; Lin, 2005; Maksymiec, 2007).

Silicon (Si) is the second most abundant element in the earth’s crust. Si nutrition alleviates various kinds of biotic and abiotic stresses (Marschner, 1995; Exley, 1998; Epstein, 1999; Ma, 2004; Balakhnina *et al.*, 2012; Farooq and Dietz, 2015). High silica contents in plant tissues, particularly in graminaceous plants such as rice, maize, or barley, activate physical and biochemical defense mechanisms for increased stress tolerance (Ma *et al.*, 2006; Currie and Perry, 2007; Mitani *et al.*, 2009*a*, *b*). Silica depositions underneath leaf cuticles form the subcuticular double layer which contributes to biotic stress resistance and reduces water loss by transpiration, thereby improving water use efficiency particularly under abiotic stresses (Lux *et al.*, 2002; Hattori *et al.*, 2005). Furthermore, decreased lipid peroxidation, reduced membrane permeabilization, and higher activities of stress defense enzymes could be linked to Si nutrition under drought, cold, and salt stress (Liang *et al.*, 2007; Farooq *et al.*, 2015). Savvas *et al.* (2008) reported increased CO_2_ assimilation rates and a substantial decrease in the uptake and translocation of sodium (Na^+^) and chloride (Cl^−^) ions into leaves of salt-stressed zucchini in the presence of Si in the growth medium. Similarly, Si deposition in the cell wall of roots correlated with immobilization of toxic metals such as aluminum (Al) in barley (Hammond *et al.*, 1995), manganese (Mn) in cucumber (Shi *et al.*, 2005), and Cd in maize (Vaculik *et al.*, 2009, 2012). Hence Si affects entry and detoxification of metal ions in the plant body. Improved antioxidative capacity and increased concentrations of ascorbate and glutathione are also suggested to explain Si-mediated metal stress tolerance (Shi *et al.*, 2005; Ma and Yamaji, 2006). These nutritional benefits support the view that Si functions as a beneficial element for plants. Its inclusion in the list of elements essential for higher plants is debatable. Most studies elucidate the importance of Si in a specific physiological and biochemical context, and for this Si is added either prior to or simultaneously with the stressor. Thus, questions as to the reversibility of stress effects by treating plants afterwards with Si but also in relation to the molecular mechanisms involved remain unanswered.

To understand the beneficial Si syndrome in its entirety we need to target and identify genes involved in signaling or regulatory pathways. Recently, a new family of genes with 18 members termed SAPs (stress-associated proteins) was identified in rice. An important role for SAPs in abiotic stress acclimation is indicated by expression profiling and transgenic approaches. SAP family members are characterized by the presence of an A20/AN1 zinc-finger domain. Proteins with such a domain are present in all eukaryotes and are also well characterized in animals (Mukhopadhyay *et al.*, 2004). Members of the SAP gene family present in rice and other plant species show specific inducibility to one or the other abiotic stresses (Vij and Tyagi, 2008; Solanke *et al.*, 2009; Giri *et al.*, 2011). Little is known about their modulation under heavy metal stress, in particular Cd toxicity. In *Arabidopsis thaliana*, expression of AtSAP10 containing multiple cysteine and histidine residues in the AN1 and A20 domains could be linked to metal binding and confers tolerance to nickel (Ni), zinc (Zn), and Mn toxicity (Dixit and Dhankher, 2011). Increasing evidence suggests that SAPs play decisive roles in stress acclimation; for example, OsSAP1 overexpression improves drought tolerance of transgenic rice (Dansana *et al.*, 2014).

To address the question of Si-induced reversibility of Cd stress effects and the suitability of SAPs as readout for stress intensity, a hydroponic study was conducted to characterize the expression profiles of 18 SAP gene family members in rice exposed to Cd stress and their respective modulation by Si application. We hypothesized that post-stress application of Si recovers growth impairment caused by Cd toxicity through altering stress-related proteins. The results will provide kinetic insight into the Si effect in plant stress tolerance and address early response mechanisms.

## Materials and methods

### Plant material and growth conditions

Seeds of rice (*Oryza sativa* L.) cv. IR64 were obtained from the International Rice Research Institute (IRRI, Los Baños, Phillipines). After surface sterilization with 5% NaOCl solution, and thorough rinsing and soaking in distilled water in darkness for 48h, the seeds were germinated on vermiculite with 0.5× Hoagland solution: 3mM KNO_3_, 0.5mM (NH_4_)H_2_PO_4_, 1mM MgSO_4_, 2mM Ca(NO_3_)_2_, 35 µM Fe-EDTA, and microelements (0.1 µM Na_2_MoO_4_, 0.32 µM CuSO_4_, 0.77 µM ZnSO_4_, 5 µM MnCl_2_, and 20 µM H_3_BO_3_) (Golldack *et al.*, 2002). After 10 d, eight uniform seedlings were selected and transferred to 5 liter plastic pots containing 0.5× Hoagland solution. Seedlings were grown for another 28 d in a growth chamber with 14h light (300 µmol m^−2^ s^−1^, 25 ^o^C) and 10h dark (21 ^o^C) with 50% relative humidity. Hydroponic solution was renewed every 5 d for the first 20 d, then every 3 d for the remainder of the experiment, and the pH was adjusted to 6.2 by using either 1M HCl or 0.5M KOH on a daily basis. At the age of 38 d, plants were stressed with 10 µM CdCl_2_ added to the nutrient solution for 8 d, while the control plants were maintained in Hoagland medium lacking Cd. Silicon treatments (0 or 0.6mM Si) were introduced 4 d after Cd stress by using sodium silicate (Na_2_O_3_Si) solution. An equivalent amount of NaCl was added to the Si-free plants to compensate for the Na content of the Na_2_O_3_Si-treated plants. The steady-state quantum yield of photosystem II (ΦPSII) was measured in atmospheric CO_2_ at 0, 12, 24, 36, 48, and 96h after Si supplementation (Mini-PAM Fluorometer, Walz, Germany) under light conditions as indicated above. Both young and mature leaves were selected randomly from each treatment and measured several times. The photosynthetic yield was calculated according to the manufacturer’s instructions. For biochemical parameters, both leaves and roots were harvested 4 d after Cd stress [i.e. prior to Si application (42 d)] and also at the end of the experiment (46-day-old plants; 4 d after Si supply), immediately frozen in liquid nitrogen, and stored at –80 ^o^C until further analyses. For assessing plant growth, roots were initially drained out between paper towels and plants were separated into leaves, shoots, and roots. Thereafter, plant tissues were dried at 65 °C to constant weight for dry biomass yield.

### Hydrogen peroxide (H_2_O_2_) quantification

Plant material for H_2_O_2_ quantification was immediately frozen in liquid nitrogen and then stored at –80 ^o^C. H_2_O was quantified as described by Pérez and Rubio (2006). Stored leaves (0.1g) were pulverized with a pestle and mortar in liquid nitrogen, and then H_2_O_2_ was extracted with 0.5ml of 5% trichloroacetic acid (TCA). The homogenate was centrifuged at 13 000 *g* for 10min. After dilution with 0.1M sodium carbonate buffer, 20 µl aliquots were incubated with 50U of catalase (bovine liver, Sigma, USA) or with the same volume of water for 10min at 30 ^o^C as control. H_2_O_2_ was determined by chemiluminescence (CL) with luminol. The sample (2 µl) was added to 1ml of reagent solution [stock luminol and stock Co(II) solution diluted in 0.1M sodium carbonate buffer, pH 10.2]. The emitted photons were counted over 7s with a luminometer (Mini Lumat LB 9506, Berthold, D-Bad Wildbad). The difference between catalase-treated and untreated samples (∆CL) was considered as H_2_O_2_-specific CL. A standard curve was generated using appropriate dilutions of 30% H_2_O_2_ (Carl Roth, Germany).

### Ascorbate

Ascorbate and dehydroascorbate (DHA) were determined as described by Horling *et al.* (2003). Leaves were pulverized in liquid N_2_ and extracted with 1ml of 1M HClO_4_. After centrifugation at 13 000rpm (5min at 4 °C), 400 μl of supernatant was transferred to 200 μl of 1M HEPES/KOH buffer (pH 7.0). The pH of the solution was adjusted to pH 5.0–6.0 with 5M K_2_CO_3_. After centrifugation, the supernatant was used for measuring the contents of reduced and total ascorbate spectrophotometrically.

Ascorbate was measured after adding 150 μl of supernatant to 850 μl of 0.1M sodium phosphate buffer (pH 5.6) by monitoring the decrease in *A*_265_ in the presence of 5U of ascorbate oxidase (Sigma, Deisenhofen, Germany). For measuring total ascorbate, DHA was reduced with 50mM DTT in four volumes of 0.1M sodium phosphate (pH 7.0) during 30min of incubation on ice and ascorbate was analyzed as described above. DHA was calculated as the difference of ascorbate contents determined in the presence and absence of DTT according to identically treated ascorbate and DHA standards.

### Glutathione and non-protein thiols

Glutathione was quantified with an enzyme-cycling assay based on oxidation of GSH by DTNB (2,2'-dinitro-5'5-dithiodibenzoic acid) and reduction of GSSG by NADPH in the presence of glutathione reductase (GR) (Griffith, 1980) with a few modifications. A 200mg aliquot of frozen plant material was extracted in 1ml of 0.1M HCl and 0.1mM EDTA. For total GSH, 200 µl of neutralized supernatant was incubated with 6mM DTNB for 5min followed by 15min incubation with 5 µl of 2-vinylpyridine. After centrifugation, the reaction was started by adding GR, and changes in DTNB absorbance were monitored at 412nm for 8min. For GSSG, the neutralized supernatant was incubated with 2-vinylpyridine for 15min followed by 5min DTNB incubation and subsequently GR and NADPH. The difference between total glutathione and GSSG contents is presented as the GSH content.

Non-protein thiols (NPTs) in leaf and root samples were determined as described by Sharma *et al.* (2004). A 0.1g aliquot of the plant material was extracted with 1ml of 1M HCl and 1mM EDTA. The extract was added to 0.8ml of assay buffer (0.12M Na-phosphate, pH 7.8) and 100 µl of 6mM DTNB. The absorbance was recorded at 412nm and compared with a calibration curve with GSH.

### Elemental analyses

Leaf sheaths, roots, and shoots (including leaf blades) were separated, and apoplastic Cd from roots was desorbed with 5mM PbNO_3_ at 4 °C for 30min. Samples were dried at 65 °C, homogenized, and microwave digested (START 1500; MLS GmbH, Leutkirch, Germany) in 2ml of 30% (w/v) H_2_O_2_ and 4ml of 65% HNO_3_ with the following temperature protocol: 12min 30s ramping to 80 °C, 5min 30s at 80 °C, 4min ramping to 180 °C, 12min at 180 °C. Plastic labware was used to prevent Si contamination. Element compositions (including Si) were determined with an inductively coupled plasma atomic emission spectrometer (ICP-AES, iCAP 6500, Thermo Scientific, Waltham, MA, USA).

### Targeted transcript analyses

Total RNA was extracted with the Trizol reagent (Life Technologies, Karlsruhe, Germany) and reverse transcribed (Wormuth *et al.*, 2006). Semi-quantitative RT-PCR was performed to optimize equal loading of cDNA using actin primers as reference (Finkemeier *et al.*, 2005). For each transcript, root cDNA from control plants was used for annealing temperature and cycle number optimization. Supplementary Table S1 at JXB online contains the list of gene-specific primers designed by Primer3Plus software (http://www.bioinformatics.nl/cgi-bin/primer3plus/primer3plus.cgi/). Quantitative real-time PCR (qRT-PCR) was then performed using the iCycler™ Thermal Cycler (Bio-Rad, USA) with the iQTM SYBR Green Supermix (Bio-Rad, USA) in a final volume of 20 µl using actin as an internal control. The standard thermal program consisted of the following steps: 95 °C for 1min; 45× (95 °C for 30s, 58 °C for 40s, 72 °C for 45s), 72 °C for 10min followed by a melting curve program (55–95 °C in increasing steps of 0.5 °C). Samples from each treatment were run in duplicate, and values in Supplementary Fig. S2 represent the average from two independent experiments. Efficiencies of each reaction were calculated using LinRegPCR software (Ruijter *et al.*, 2009). The relative expression level was calculated as values relative to corresponding control samples at the indicated time points, after normalization to actin and α-tubulin using the threshold cycles (average background subtracted) according to the equation of Pfaffl (2001).

### Statistical analysis

The data were subjected to statistical analysis by using the *t*-test, and treatments were compared by calculating means with SD at *P*≤5%.

## Results

### Plant growth and photosynthetic response

**Fig. 1.**
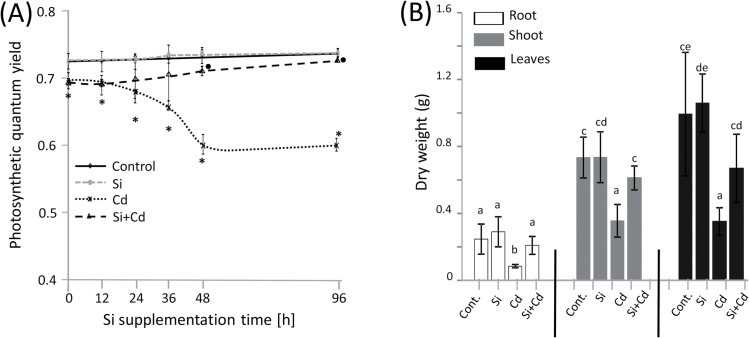
Plant growth and photosynthetic characteristics of rice genotype IR64 grown in hydroponic nutrient solution with or without Cd and supplementary Si. (A) Kinetic changes of photosynthetic quantum yield (ΦPSII) of rice treated with Cd for 4 d prior to inititation of recovery by addition of Si (*t*=0h). Also depicted are untreated control, and single treatments with Cd and Si. Significant differences between Cd-treated and control rice are denoted by an asterisk, while a filled circle indicates a significant difference between Cd/Si-treated and Cd-treated plants. (B) Dry weight of rice roots, shoots, and leaves recorded at harvest (46-day-old plants). Data groups of significant difference are labeled with different letters (Student’s *t*-test, *P*<0.05). The data are means ±SD of *n*=108 (A) and *n*=3 (B) from three independent experiments.

Thirty-eight-day old IR64 rice plants were treated with 10 µM CdCl_2_ for 4 d prior to addition of 0.6mM Si. In order to test the hypothesis that Si supplementation enables recovery from established Cd stress, ΦPSII was determined at regular time intervals between 0h and 96h. Both young and old leaves from each treatment were measured several times. Differences were not detected between control plants grown with and without Si supply (Fig. 1A). The 4 d period of Cd exposure had significantly decreased the ΦPSII, indicating progressing Cd toxicity. The decline continued until a minimum value was recorded 48h after onset of measurement (6 d after Cd addition) and remained at this level during the next 2 d. The minimum yield under Cd stress was ~21% lower than in control plants. Contrastingly, photosynthetic efficiency of Cd-treated plants receiving Si nutrition increased with time and reached the ΦPSII of untreated controls after 96h and hence was ~18% higher than in stressed plants without an extra Si supply.

Further, the plants were investigated for growth performance (Fig. 1B). Growing rice for 8 d on nutrient solution supplemented with 10 µM Cd severely inhibited growth of all tissues. Roots most sensitively responded to Cd. Thus, root biomass of Cd-treated plants was 35% of that of the control, while Cd-treated shoots and leaves reached 40–50% that of untreated controls. Si supplementation substantially ameliorated the growth inhibition by Cd. Dry biomass of Cd-treated plants was 1.7- to 2.4-fold higher in the presence of Si compared with Cd-stressed plants lacking Si (Fig. 1B), while there was no significant difference between control plants supplied or not with Si.

### Hydrogen peroxide and ascorbate contents

**Fig. 2.**
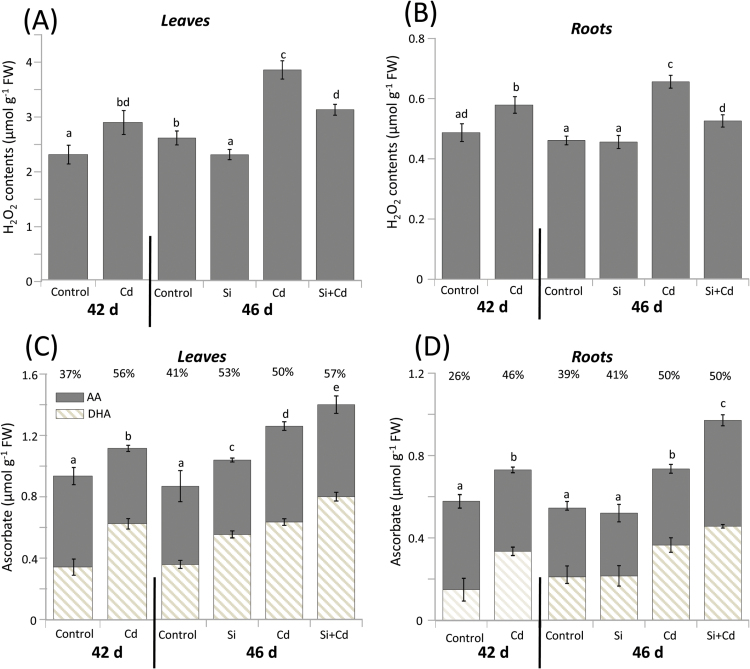
Hydrogen peroxide and ascorbate levels dependent on Cd and Si treatment of rice. Leaf (A, C) and root (B, D) contents of H_2_O_2_ (A, B) and ascorbate (C, D) were determined in 42-day-old plants stressed with Cd for 4 d prior to Si application and subsequently treated with Si for another 4 d (labeled as 46-day-old plants), with appropriate controls. In (C and D), contents of ascorbate in its reduced (AA; solid) and oxidized (DHA; hatched) form are given. The percentage values above the columns represent the oxidation state of the ascorbate pool. The data are means ±SD from three independent experiments and *n*=18 (A and B) and *n*=6 (C and D) determinations. Data groups of significant difference were calculated by *t*-test and are labeled with different letters (*P*<0.05).

As measure of oxidative stress, H_2_O_2_ was quantified in both leaves and roots. Compared with control plants, the 4 d Cd stress before Si application caused a significant increase in H_2_O_2_ contents in leaves and roots by 22% and 26%, respectively (Fig. 2A, B). H_2_O_2_ accumulation in leaves and roots increased further during the next 4 d of Cd exposure, while Si supplementation to Cd-stressed plants reversed the Cd-induced H_2_O_2_ accumulation to a large extent. However, H_2_O_2_ contents in non-stressed Si-supplemented plants were marginally lower in leaves and unaltered in root tissues, when compared with control plants (Fig. 2A, B).

Ascorbate levels were analyzed as a major low molecular mass antioxidant linked to redox homeostasis. The ascorbate and DHA levels significantly increased in leaves and roots during the first 4 d of Cd exposure (Fig. 2C; 42 d) and in the subsequent 4 d until 46 d. Leaf ascorbate levels of control plants were slightly increased after Si application. However, the most pronounced increase in ascorbate levels was recorded when Si was applied to Cd-stressed rice. The oxidation state of the ascorbate pool ranged between 37% and 57%, and, importantly, the upper range of oxidation was noticed under combined application of Cd and Si, suggesting involvement of Si in modulating ascorbate homeostasis. Root total ascorbate response was significant when plants either experienced Cd toxicity alone or were supplemented with Si under Cd stress (Fig. 2D). As compared with controls, ascorbate levels reached 127% and 137% after 4 d and 8 d of Cd stress, respectively (Fig. 2D). In the presence of Si and Cd, the size of the ascorbate pool reached up to 176% as compared with untreated control plants. Again, the oxidation level was higher in the Cd-treated plant material.

### Glutathione and non-protein thiol contents

**Fig. 3.**
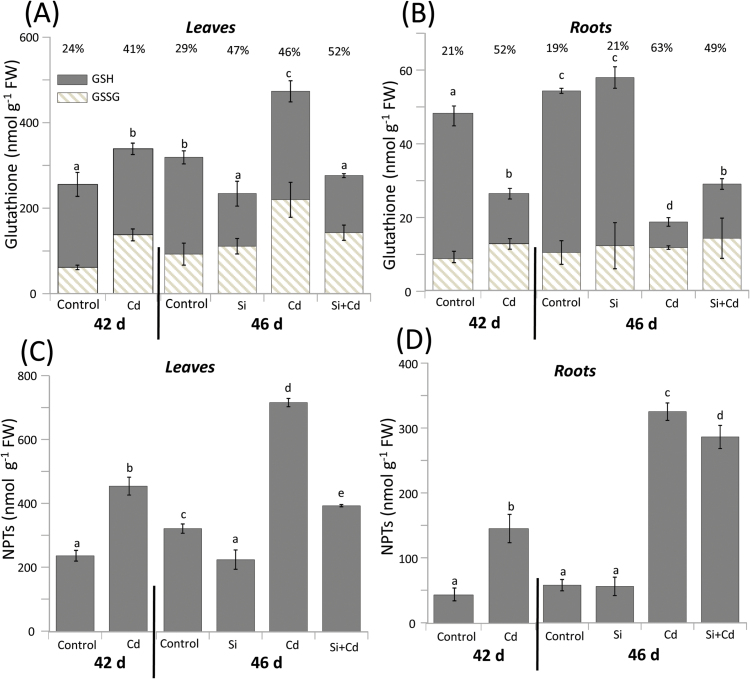
Glutathione and non-protein thiol levels dependent on Cd and Si treatment of rice. Leaf (A, C) and root (B, D) contents of glutathione (A, B) and non-protein thiols (NPTs) (C, D) were measured in 42-day-old plants stressed with Cd for 4 d prior to Si application and subsequently treated with Si for another 4 d (labeled as 46-day-old plants), with appropriate controls. In (A and B), contents of glutathione in its reduced (GSH; solid) and oxidized (GSSG; hatched) form are presented, as is the oxidation state as a percentage above the columns. The data are means ±SD from three independent experiments and *n*=6 (A and B) and *n*=15 (C and D) determinations. Data groups of significant difference were calculated by *t*-test and are labeled with different letters (*P*<0.05).

The response pattern of glutathione differed greatly from that of ascorbate. Leaf glutathione levels were 30% increased after 4 d of Cd exposure (42 d) and 50% after 8 d (Fig. 3A). Interestingly, in Cd-stressed roots, total glutathione was 43% below that of control roots after 4 d and 70% after 8 d (Fig. 3B). Si application alone decreased the total glutathione contents by 27% in leaves, while it was ineffective in roots (Fig. 3A, B). Si reversed the contrasting Cd effects in leaves and roots (i.e. decrease in leaves and increase in roots; Fig. 3A, B). The proportion of oxidized glutathione ranged between 19% and 63%. The highly oxidized state was observed in the Cd-stressed roots.

Phytochelatin synthesis represents the major Cd detoxification mechanism in plants (Clemens and Persoh, 2009; Rea, 2012). The possible impact of Si on Cd chelation by phytochelatins was addressed by quantifying NPTs. Subtracting glutathione from NPT gives a reasonable estimate of phytochelatins. Roots responded to Cd exposure more strongly than leaves, with an ~3.3- and 5.6-fold increment in NPTs after 4 d and 8 d stress, respectively (Fig. 3D). In Cd-treated plants, leaf NPT contents were ~1.9-fold higher after 4 d, and 2.2-fold after 8 d exposure than in control leaves (Fig. 3C). The response strength in roots of Cd-treated plants slightly decreased upon Si supplementation, but decreased by 45% in leaves which compensated for ~80% of the Cd-induced effect (Fig. 3C, D). Si supplementation to control plants slightly decreased leaf NPTs, while no change occurred in roots.

### Elemental composition

**Fig. 4.**
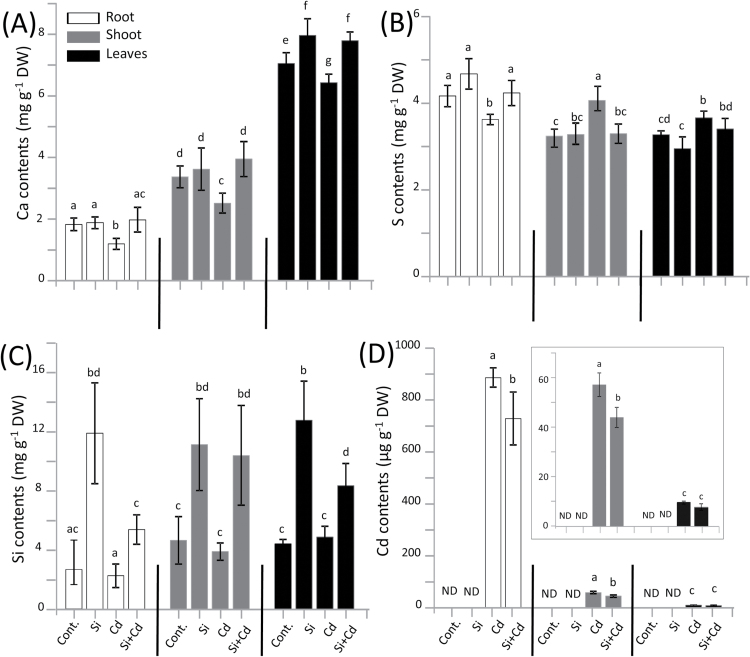
Calcium (Ca), sulfur (S), silicon (Si), and cadmium (Cd) contents in roots, shoots, and leaves of rice genotype IR64 grown in hydroponic nutrient solution with or without Cd and supplementary Si. Data are means ±SD (*n*=4) from four independent experiments. ND, not detected. Data groups of significant difference were calculated by *t*-test and are labeled with different letters (*P*<0.05).

Heavy metal accumulation and compartmentation represent decisive parameters in heavy metal tolerance. The elemental composition in roots, shoots, and leaves of Cd-stressed rice varied significantly between plants grown in the presence or absence of a Si supply (Fig. 4; Supplementary Fig. S1). As expected, tissue Si contents were substantially elevated following their addition to the hydroponic culture (Fig. 4C). Interestingly, a major proportion of Si applied under stress was translocated to above-ground rice tissues. Cd was undetectable in control plants. Cd accumulated less in roots in the presence of Si. Also 24% less Cd was translocated to shoots (Fig. 4D). In contrast, mean Cd contents of leaf blades were insignificantly lower following Si supply. Furthermore, the presence of Cd in the growth medium significantly lowered the accumulation of essential macro- and micronutrients such as calcium (Ca), potassium (K), magnesium (Mg), and Zn (Fig. 4A; Supplementary Fig. S1). This effect was significantly dampened by the inclusion of Si in the nutrient formulation of stressed plants. For instance, root Ca accumulation was improved to control levels, enabling higher root/shoot translocation and similarly 21% more Ca accumulated in leaves of Cd-stressed rice receiving Si supply (Fig. 4A). Similar but smaller effects were seen for K, Mg, and Zn contents (Supplementary Fig. S1). In contrast, Cd caused an increase of leaf and shoot S contents by 12% and 25%, respectively (Fig 4B). The addition of Si lowered the shoot S contents in the presence of Cd. The root S response was opposite, with 13% less S accumulation under Cd stress. This effect was partly reversed upon Si addition as indicated by the 17% increase (Fig. 4B).

### Transcript analyses

**Fig. 5.**
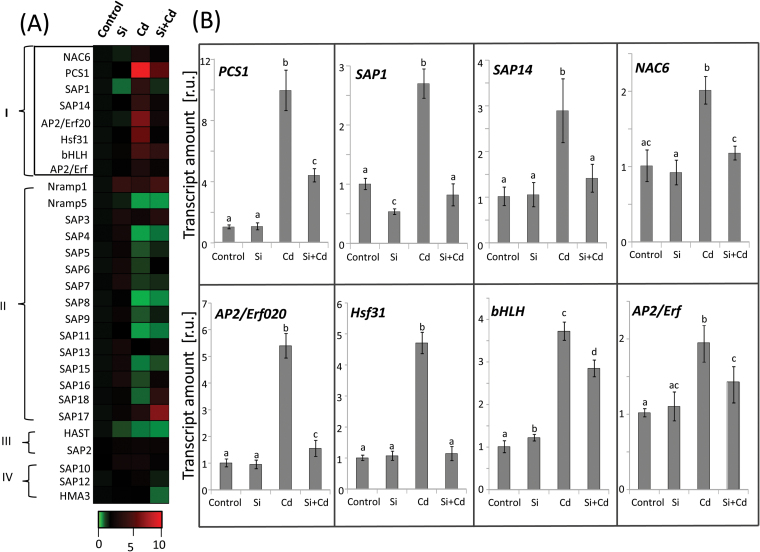
Response pattern of transcripts in rice roots grown with or without Cd and supplementary Si. (A) Expression profiles of transcripts 4 d after Si supply represented as a heat map. Based on their differential response to Cd and Si, transcripts were classified into four groups (see text for details, and Supplementary Fig. S2 with Group II–Group IV category members). In (B), the transcriptional response of targets following the recovery pattern (Group I) are shown. Data are means ±SD (*n*=6) from three independent experiments. Data groups of significant difference were calculated by *t*-test and are labeled with different letters (*P*<0.05). (This figure is available in colour at *JXB* online.)

**Fig. 6.**
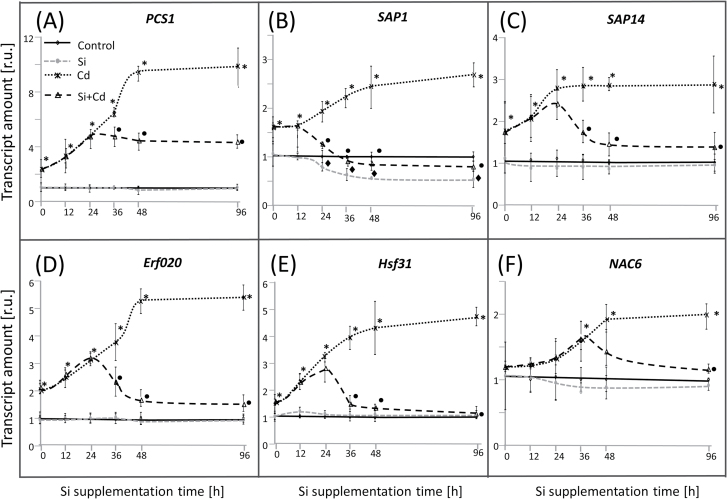
Time course analysis of transcript regulation for selected targets in roots of control and Si-supplied rice plants with or without Cd exposure. mRNA levels were quantified by qPCR from three independent experiments with duplicate determinations. Data are means ±SD, asterisks denote significant differences between Cd-treated and control rice, filled circles indicate a significant difference between Cd/Si-treated and Cd-treated plants, and filled diamonds mark significant differences between Si-treated and control plants. Student’s *t*-test, *P*<0.05.

The redox state-related data described so far indicated a high efficiency of Si in reversing Cd-induced disturbances of biochemical homeostasis. Transcript analysis was performed to identify markers of Si-mediated alleviation of Cd stress. We focused on genes encoding the 18 members of stress-inducible SAPs (Vij and Tyagi, 2006), Cd-related metal transporters, and detoxification mechanisms, namely phytochelatin synthase (*PCS1*, LOC_Os05g34290.1), *Nramp1* (LOC_Os07g15460.1), *Nramp5* (LOC_Os07g15370.1), *HMA3* (LOC_Os07g12900.1), and *HAST* (LOC_Os03g09970.1) (Ogawa *et al.*, 2009; Miyadate *et al.*, 2010; Takahashi, 2011; Sasaki *et al.*, 2012; Zhou *et al.*, 2014). Additionally, Cd-responsive transcription factor (TF) genes, namely *NAC6* (LOC_Os03g60800.1)], *AP2/Erf020* (LOC_Os05g34730.1), *Hsf31* (LOC_Os02g32590.1), *bHLH* (LOC_Os01g06640.1), and *AP2/Erf* (LOC_Os07g22730.1), were analyzed in order to identify signaling components potentially involved in Cd toxicity and Si/Cd antagonism. These TFs were selected from two previous experiments with Cd-stressed rice roots where transcriptomes had been profiled using gene chips and RNA-seq (http://genevestigator.com/gv/; Hruz *et al.*, 2008; Ogawa *et al.*, 2009; He *et al.*, 2015). The transcripts were quantified 4 d after Si supply. Obtained response patterns to Cd and/or Si were categorized into four groups: (I) antagonistic effects of Si on Cd toxicity (Fig. 5B); (II) independent effects of Si and Cd; (III) additive effects (positive or negative) of Si and Cd; and (IV) complex patterns (Fig. 5A; Supplementary Fig. S2). Six promising targets from group I (Fig. 5B) showing the Si-induced reversal of the Cd effect were selected for a time course analysis until 96h after Si addition in order to describe the recovery phase initiated by Si supplementation. *PCS1* was selected as a marker for Cd stress and revealed an ~10-fold up-regulation at *t*=48h which was unchanged until the end at day 4. In the presence of Si, the changes first followed the kinetics observed in the Cd-treated sample until 24h, but then started to decline without reaching the value of the non-stressed control (Fig. 6A). A similar reversal pattern was detected for the expression of both TF genes. *AP2*/*Erf020* was induced 5.4-fold, while *Hsf31* revealed a 4.7-fold up-regulation at *t*=96h as compared with the control (Fig. 6D, E). In contrast, in the presence of Si under Cd stress, the *Hsf31* transcript level dropped already after 36h, near to the level of the control (Fig. 6E). The *AP2/Erf020* accumulation in Si/Cd plants first followed the kinetics observed in the Cd plants until 24h, then slightly declined at *t*=36h, and subsequently reached the control level (Fig. 6D). *SAP1* and *SAP14* transcript levels also increased in parallel in Cd and Cd/Si tissue, however only for 12h. Si supplementation reversed the Cd effect almost completely (Fig. 6B, C). The maximal delay was detected for *NAC6* mRNA (Fig 6F). Following the parallel increase in mRNA in the Cd and Cd/Si plants until 36h, *NAC6* levels continued to rise in the Cd-stressed sample, while they decreased to levels close to the control in the Cd/Si sample (Fig. 6F). For all transcripts, extra Si supply to control plants caused no change in mRNA levels, except for *SAP1* which was down-regulated by almost 2-fold as compared with the respective control (Fig. 6B).

## Discussion

### Avoidance and repair as a combined strategy for Si-induced reversibility of Cd toxicity

Si modulates tolerance levels to biotic and abiotic stresses and also ameliorates heavy metal toxicity (Nwugo and Huerta, 2008; Kaya *et al.*, 2009). Si-dependent recovery from pre-established stress conditions has not been analyzed in detail. This experimental design promises access to a kinetic and mechanistic understanding. The first two questions addressed in this work concerned the appropriate experimental design and the correctness of the hypothesis that Si administered post-stress ameliorates the negative effects of pre-established Cd toxicity.

Cd added to the hydroponic medium significantly reduced ΦPSII after 4 d of treatment and therefore clearly established Cd toxicity effects which were further aggravated until 8 d (Fig. 1A). Photosynthesis is also a known sensitive target of Cd toxicity in rice (Moya *et al.*, 1993). In contrast, Si supply prevented further development of damage and improved ΦPSII within 48h, reaching maximal values close to those of non-stressed plants. Apparently Si application after onset of Cd stress allowed for recovery of the rice plants. Previously, Si was shown to reduce the inhibitory effects of Cd on the photosynthetic machinery of cucumber by increasing the contents of photosynthetic pigments and reducing the damage to thylakoid membranes (Feng *et al.*, 2010). In a recent screening for dominant changes in the rice leaf proteome, Nwugo and Huerta (2011) identified 60 proteins that were differentially regulated in response to Cd treatment in plants lacking or pre-treated with Si. With a 30% share, polypeptides with functions in photosynthetic processes represented the largest functional category among the identified proteins. This study goes beyond our present knowledge since the photosynthetic performance of Cd-stressed rice recovered by post-stress application of Si.

H_2_O_2_ as a typical Cd toxicity symptom accumulated in leaves and roots after 4 d. The accumulation was significantly reduced in Si-treated plants (Fig. 2A, B). Ascorbate and glutathione play decisive roles in cell redox homeostasis, antioxidant defense, and plant development in normal metabolism and under stress (Mittler *et al.*, 2004; Dowdle *et al.*, 2007; Schlaeppi *et al.*, 2008; Mhamdi *et al.*, 2010). The results indicate a lowered oxidative load of Cd-stressed rice in the presence of Si (Figs 2C, D, 3A, B). The major difference between ascorbate and glutathione response was that ascorbate levels further increased in both leaves and roots under Si/Cd treatment beyond the already elevated levels found in Cd-treated rice. The enlarged ascorbate pool size indicates stimulated defense capacity by providing additional substrate for the water–water cycle, and for quenching of the tocopherol radical and ROS, particularly H_2_O_2_ (Song *et al.*, 2009; Gest *et al.*, 2013). This type of positive Si effect on the water–water cycle was described in the context of salinity stress and mycorrhization (Garg and Bhandari, 2016). NTPs under non-stress conditions tentatively match the glutathione pool (Metwally *et al.*, 2005) as also seen in this study (Fig. 3). As previously reported for barley (Finkemeier *et al.*, 2003), Cd stress reduced root contents of glutathione which was converted to phytochelatins (Fig. 3B). This is apparent from the greater difference between NPTs and glutathione which reached >244 nmol g^–1^ FW in leaves, and 306 nmol g^–1^ FW in roots after 8 d exposure to Cd stress. The value dropped to 118 nmol g^–1^ FW in leaves and 257 nmol g^–1^ FW in roots from Cd/Si plants. The Si-induced drop in phytochelatin-bound thiols indicates decreasing concentrations of free Cd in the cytosol. Phytochelatins mediate sequestration into the vacuole. For this study, it is important that Si supply to Cd-stressed plants significantly increased the glutathione proportion within the NPT pool of roots. It is concluded that lower Cd levels in the cytoplasm reduced the PCS1 activity and the drainage of GSH into phytochelatin synthesis (Fig. 3B). The recovery experiment advances the study of Song *et al.* (2009) who reported similar increases in the glutathione pool of Cd-stressed Brassica plants when Si and Cd were supplied simultaneously. Apparently Cd-induced phytochelatin synthesis drained more glutathione than could be synthesized in roots, leading to a >5-fold increase in NPTs. The opposite pattern in leaves, namely up-regulation of glutathione in Cd-treated tissue and down-regulation upon Cd/Si treatment, indicates that stimulation of glutathione synthesis was able to overcompensate for the drainage into phytochelatin synthesis. Song *et al.* (2009) reported synergistic effects of simultaneous Cd and Si supply on glutathione contents of *Brassica chinensis*. The discrepancies might be caused by the entirely different experimental design and distinct nutrient solution, as well as species differences.

The elemental analysis revealed reduced Cd accumulation in roots in the presence of Si (Fig. 4). Cd is first absorbed apoplastically and then transported across the plasma membrane with the help of secondary cation transporters such as *IRT1* (Clemens *et al.*, 2002; Clemens, 2006). Except for Cd-hyperaccumulating species, Cd accumulates more in roots than in shoots and leaves, which is in line with our observations (Kirham, 2006). Si uptake is an active process particularly in silica-accumulating species such as rice (Ma *et al.*, 2006). Si strongly binds to cell wall components and contributes to cross-linking of cell wall structures. Si-induced structural alterations and blockage of the apoplasmic transport route are suggested to reduce Cd uptake by roots and translocation to shoots (Liang *et al.*, 2001, 2007; Lukacova *et al.*, 2013). However, Cd concentrations in leaves did not differ significantly between plants with or without Si. Apparently substantial Cd amounts had already accumulated during the first 4 d of Cd exposure before the start of the Si treatment. Thus, Si-dependent blockage of uptake and transfer of Cd from roots to shoots cannot explain the recovery of photosynthesis in the recovery experiment. About 80% of the Si absorbed under Cd stress was transferred to shoots and leaves. As a consequence, Si levels were highly similar in different tissues of Si-treated control plants (Fig. 4). This suggests that the positive effects of Si administration on above-ground tissue are caused by local effects of Si accumulating in the shoot and not by effects on long-distance Cd transport. Enhanced defense as described above for ascorbate and compatible compartmentation, for example in the vacuole, are probably involved in the process.

Recently, Pavlovic *et al.* (2013) reported that Si alleviates Fe deficiency in cucumber at two mechanistic levels, namely indirectly by increasing root apoplastic iron (Fe) uptake and directly by modulation of strategy-I-responsive genes involved in synthesis of Fe-mobilizing compounds. Controlled uptake, long-distance transport, and utilization of the functionally important micro- and macroelements plays crucial roles in maintaining optimal metabolism, plant growth, and high productivity. Element analyses suggest that differential Ca translocation in the presence or absence of Cd and Si contributes to the protection of the photosynthetic apparatus. However, it should be noted that Ca contents do not equate to Ca concentrations. Ca is known to alleviate Cd toxicity (Suzuki, 2005). In addition, the repressed delivery of other elements such as Mg, K, Zn, and S was also partially improved by Si supply under Cd stress. Improved utilization of micronutrients (Fe and Zn) and macronutrients (Ca, Mg, and K) upon Si supply to chromium (Cr)- and Cd-stressed plants was previously reported by Tripathi et al. (2012a, b). However, in their study, Cd and Si were supplied simultaneously, thus recovery was not a point of interest. Here it is shown that already established negative effects of Cd are reversed upon Si supplementation. This is important since it indicates the action of mechanisms beyond uptake in roots and long-distance transport. Apparently, Si also facilitated detoxification of Cd that had already been incorporated during the first 4 d. The results also exclude a major function for Si in developmental processes underlying the Cd acclimation response, since the effects were observed in adult leaves.

### Si-mediated regulation of Cd-induced gene expression

The biochemical data provided compelling evidence for the beneficial effects of Si on the physiology of Cd-stressed rice. The response was further characterized by transcript analyses in order to identify genes involved in signaling or regulatory pathways. Transcript regulation provides fast, sensitive, and specific readouts of altered signaling pathways (e.g. as fast as 20s after light shift experiments; Moore *et al.*, 2014). The quantitative response of transcripts belonging to the SAP family and other Cd stress markers including TFs/signaling molecules could be grouped into four response patterns, among which the antagonistic effect seen for *PCS1* *SAP1*, *SAP14*, *NAC6*, *AP2/Erf020*, *Hsf31*, *bHLH*, and *AP2/Erf* most tightly followed and thus confirmed the hypothesis of reversibility of stress effects by post-stress addition of Si. The kinetics of biochemical and molecular changes as monitored for *PCS1*, *SAP1*, *SAP14*, *NAC6*, *AP2/Erf020*, and *Hsf31* transcripts gave additional insight into the Si/Cd interference. Levels of each of the six transcripts increased in response to Cd, but the distinct bifurcation kinetics of the response curves of Cd-treated plants with and without Si hints at different sensitivity thresholds, signaling pathways, and involved mechanisms (Fig. 6). Levels of none of the transcripts differed within the first 12h after Si addition.

*SAP1*, *SAP14*, and *Hsf31* in Cd and Cd/Si treatments diverged already within 24h; in fact, *SAP1* significantly more than *SAP14* and *Hsf31* (Fig. 6B, C). SAP1 and 14 belong to the recently identified SAP gene family with 18 members in rice. Overexpression of SAPs in rice and other plant species confers tolerance to various abiotic stresses. For example, Mukhopadhyay *et al.* (2004) reported the up-regulation of *OsSAP1* upon mechanical injury, submergence, abscisic acid treatment, drought, salt, and cold stress. Similarly, *OsSAP9* was found to be involved in stress response against cold, heat, and oxidative stress (Huang *et al.*, 2008). Their involvement in response to multiple stresses has been reported also for, for example, maize, Arabidopsis, tomato, and banana (Jin *et al.*, 2007; Vij and Tyagi, 2008; Solanke *et al.*, 2009; Giri *et al.*, 2011). The stimulatory action of Cd on *SAP1* and *SAP14* probably indicates a role in Cd-related stress response, and the reversal by Si in parallel with improved growth tentatively underscores this hypothesis (Fig. 6B, C). *SAP1* and *SAP14* have been reported to be up-regulated under cold, salt, and dehydration stress (Vij and Tyagi, 2006). Their early response indicates that the involved signaling pathway is linked to a rather fast and possibly linearly Cd-dependent stress-sensing mechanism such as deviation from redox and ROS homeostasis. Si would then support re-establishment of redox homeostasis.

Phytochelatins synthesized from GSH have long been known for their role in heavy metal binding and detoxification (Finkemeier *et al.*, 2003; Yadav, 2010). Therefore, up-regulation of *PCS1* is a rather specific response to Cd toxicity. The Cd-induced up-regulation of *PCS1* was reversed after 36h of Si supplementation (Fig. 6A). In Arabidopsis, Khandekar and Leisner (2011) found that the relative expression of *PCS1* in leaves is enhanced by Si under Cu stress as compared with stressed plants lacking a Si supply, while expression of the metallothionein gene *MT1a* is down-regulated in the simultaneous presence of Si and Cu. Thus there exist species-specific differences. It may be speculated that Si activated a more efficient compartmentalization and detoxification of Cd. The element analyses support this scenario (Fig. 4). On the other hand, Si supplementation stopped the further accumulation of *PCS1* transcript but failed to lower it to the control level. This may either indicate a long half-life of the *PCS1* transcript or sustained Cd stress in the cytosol.

In order to address potential signaling elements and regulators, we selected five Cd-responsive TFs, namely AP2/Erf020, AP2/Erf, bHLH, NAC6, and Hsf31, from rice transcriptome analyses, and subsequently focused on three of them which revealed an almost complete reversal upon Si application to Cd-stressed rice. He *et al.* (2015) reported up-regulation of *AP2/Erf020*-TF under Cd stress. Likewise a genome-wide transcriptome analysis in Arabidopsis revealed strong (>30-fold) up-regulation upon Cd stress of *AP2/Erf019*-TF (At1g22810), a close homolog of *AP2-Erf020* in rice (Weber *et al.*, 2006). Further, Hsf31 was chosen because its Arabidopsis homolog HsfA3 (At5g03720) was implicated in controlling the expressional up-regulation of ascorbate peroxidase 2. This mechanism was suggested to promote tolerance to oxidative stress in Arabidopsis (Hwang *et al.*, 2012). *Hsf31* significantly responded to the Si supplementation within 36h and returned to the level of the Cd-free control plants within 2 d. *Hsf31* is a member of the antioxidant regulatory network. The rapid and complete reversal of *Hsf31* transcript to the control level with Si probably indicates efficient readjustment of redox homeostasis.

The most delayed bifurcation was seen for Os*NAC6* (Fig. 6F). *OsNAC6* is drought induced and targets downstream stress-responsive genes such as apetala 2-TFs, Zn-finger proteins, and MYB TFs. Its overexpression enhances drought and salt stress tolerance in transgenic rice (Rachmat *et al.*, 2014). The late response of *NAC6* may tentatively be explained by ionic or osmotic imbalances under Cd stress (Hassan *et al.*, 2009) which may be reversed by Si only slowly. Si is known to affect drought tolerance, for example in sorghum by increased silicification or altering internal barriers (Hassan *et al.*, 2009).

Si effects on regulation of metal transporters in higher plants have only been addressed in a few studies such as that of Li *et al.* (2008) who found that Si application under Cu stress reduced the activity of *HMA5* in roots of Arabidopsis. In our study, the response of metal transporters to Cd and Cd/Si treatments was barely distinguishable, except for *OsHMA3* which is a member of heavy metal ATPases (Fig. 5A; Supplementary Fig. S2). Here, expression of *HMA3* in roots of Cd-stressed rice was not different from that in controls which tentatively fits a report by Kim *et al.* (2014) who found a significant up-regulation of *HMA3* in roots of Cd-stressed rice only during the early stress period at day 1, but the difference largely disappeared during extended exposure. Here, we observed that the expression of *HMA3* was significantly suppressed due to the presence of Si under Cd stress, which indicates that Si might be involved in Cd sequestration; therefore, less *HMA3* would be needed. This finding is in line with our results from element analyses where we found reduced Cd levels in roots associated with Si application.

In conclusion, the synergistic and antagonistic responses of H_2_O_2_, metabolites, and element levels, as well as *PCS*, *SAP* genes, *AP2/Erf020*, *Hsf31*, *NAC6*, and transporter transcripts to Cd and Cd/Si supplementation, and the distinct kinetics of the antagonistic response indicate that Si interferes with Cd stress via several mechanisms. Decreased Cd uptake and translocation, as well as improved compartmentation, readjustment of redox homeostasis, and strengthened antioxidant capacity as indicated by elevated ascorbate levels contribute to the Si effect. Among the members of the SAP family, SAP1 and SAP14 are promising candidates for involvement in the Cd toxicity response, while SAP3, 4, 5, 6, 7, 9, 13, 15, and 16 responded to Si alone and thus might be of interest for further consideration in general stress responses and Si-dependent stress amelioration. Recently, overexpression of OsSAP1 in rice was shown to improve water stress tolerance (Dansana *et al.*, 2014). The overexpressing rice lines revealed alterations in their transcriptome, with many transcripts assigned to the gene ontology group of stress-responsive genes. These findings support the conclusion that the up-regulation of *SAP1* in Cd-stressed rice and the reversal of this effect by Si indicate efficient stress relief by Si supplementation. The Si-induced recovery of Cd-stressed rice will allow for identifying early signaling responses by, for example, transcriptome profiling in the future.

## Supplementary data

Supplementary data are available at *JXB* online.

**Figure S1**. K, Mg, and Zn contents in rice genotype IR64 grown in hydroponic nutrient solution with or without Cd and supplementary Si.

**Figure S2**. Transcript abundance in roots exposed to Cd toxicity and changes in response 96h after Si supply.

**Table S1**. Sequence of primers used for real-time PCR analysis.

Supplementary Data

## Abbreviations:

bHLH: basic helix–loop–helix
Cd: cadmium
DHA: dehydroascorbate
DTNB: 2,2'-dinitro-5'5-dithiodibenzoic acid
ERF: ethylene response factor
GSH: reduced glutathione
GSSG: oxidized glutathione
Hsf: heat shock transcription factor
NPT: non-protein thiol
ΦPSII: quantum yield of photosystem II
ROS: reactive oxygen species
SAP: stress-associated protein
Si: silicon
TF: transcription factor.
