# Supplementary material for: Silicon-induced reversibility of cadmium toxicity in rice
Source: J Exp Bot. 2016 Apr 27;67(11):3573–85. doi: 10.1093/jxb/erw175 (PMC4892736; doi:10.1093/jxb/erw175)
Supplement: Supplementary Data [file supp_67_11_3573__index.html]

Silicon-induced reversibility of cadmium toxicity in rice — Silicon-induced reversibility of cadmium toxicity in rice — Supplementary Data 

# Silicon-induced reversibility of cadmium toxicity in rice

## Supplementary Data

Data files

- Supplementary\_figures\_S1\_S2\_table\_S1.pdf - Supplementary Data
