# Supplementary material for: Silicon-induced reversibility of cadmium toxicity in rice
Source: J Exp Bot. 2016 Apr 27;67(11):3573–85. doi: 10.1093/jxb/erw175 (PMC4892736; doi:10.1093/jxb/erw175)
Supplement: Supplementary Data [file supp_erw175_Supplementary_figures_S1_S2_table_S1.pdf]

Farooq et al.
(Supplementary Figure S1)

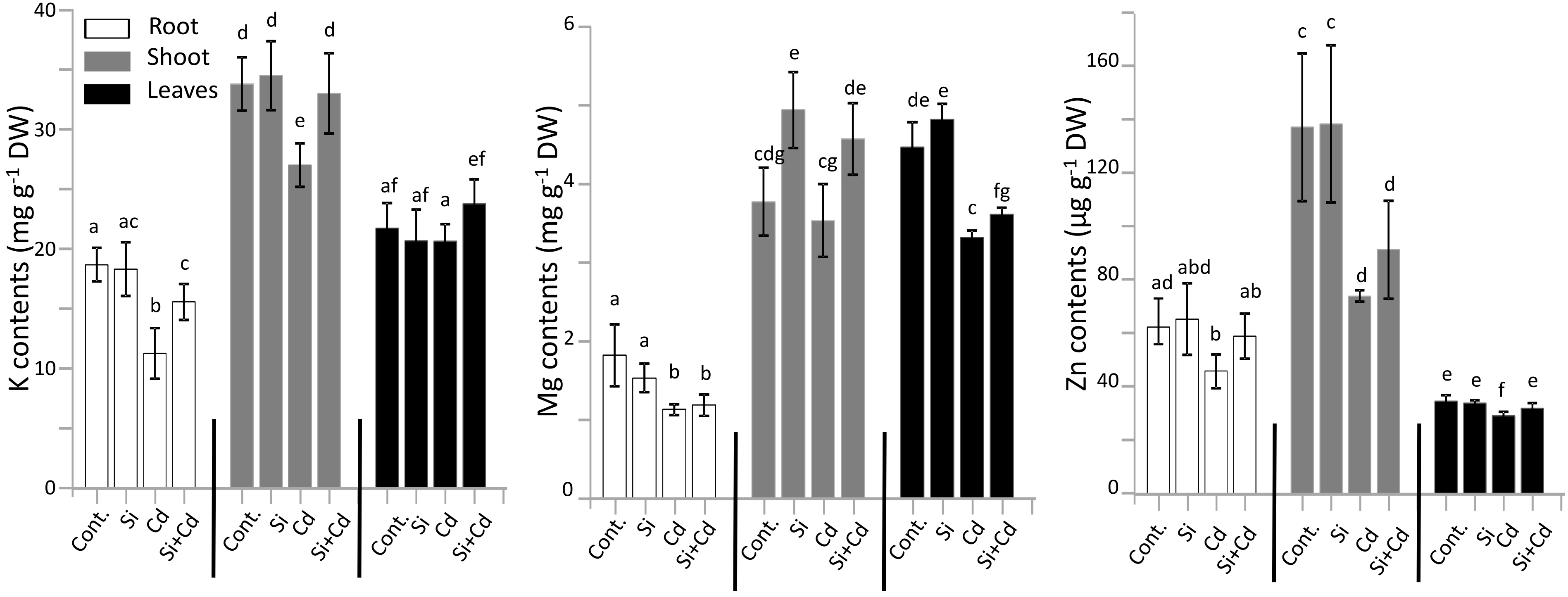

Figure S1: Potassium (K), magnesium (Mg) and zinc (Zn) contents in roots, shoots and leaves of rice genotype IR64 grown in hydroponic nutrient solution with or without Cd and supplementary Si. Data are means ± SD from four independent experiments. Data groups of significant difference were calculated by t-test and are labelled with different letters ($p<0.05$).

Supplementary Figure S2

Group II: Independent effect of Si and Cd

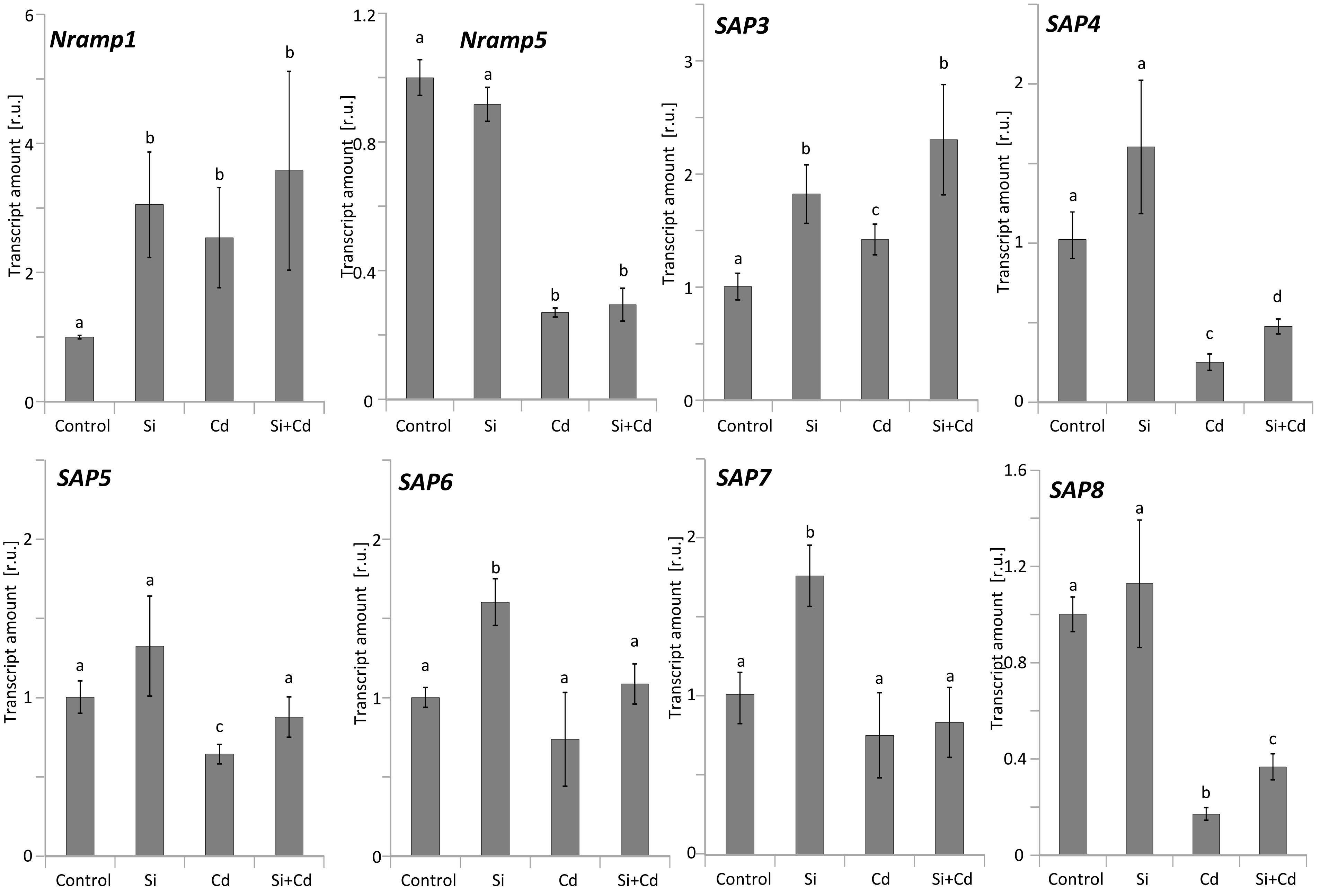

….*Group II:* Independent effect of Si and Cd

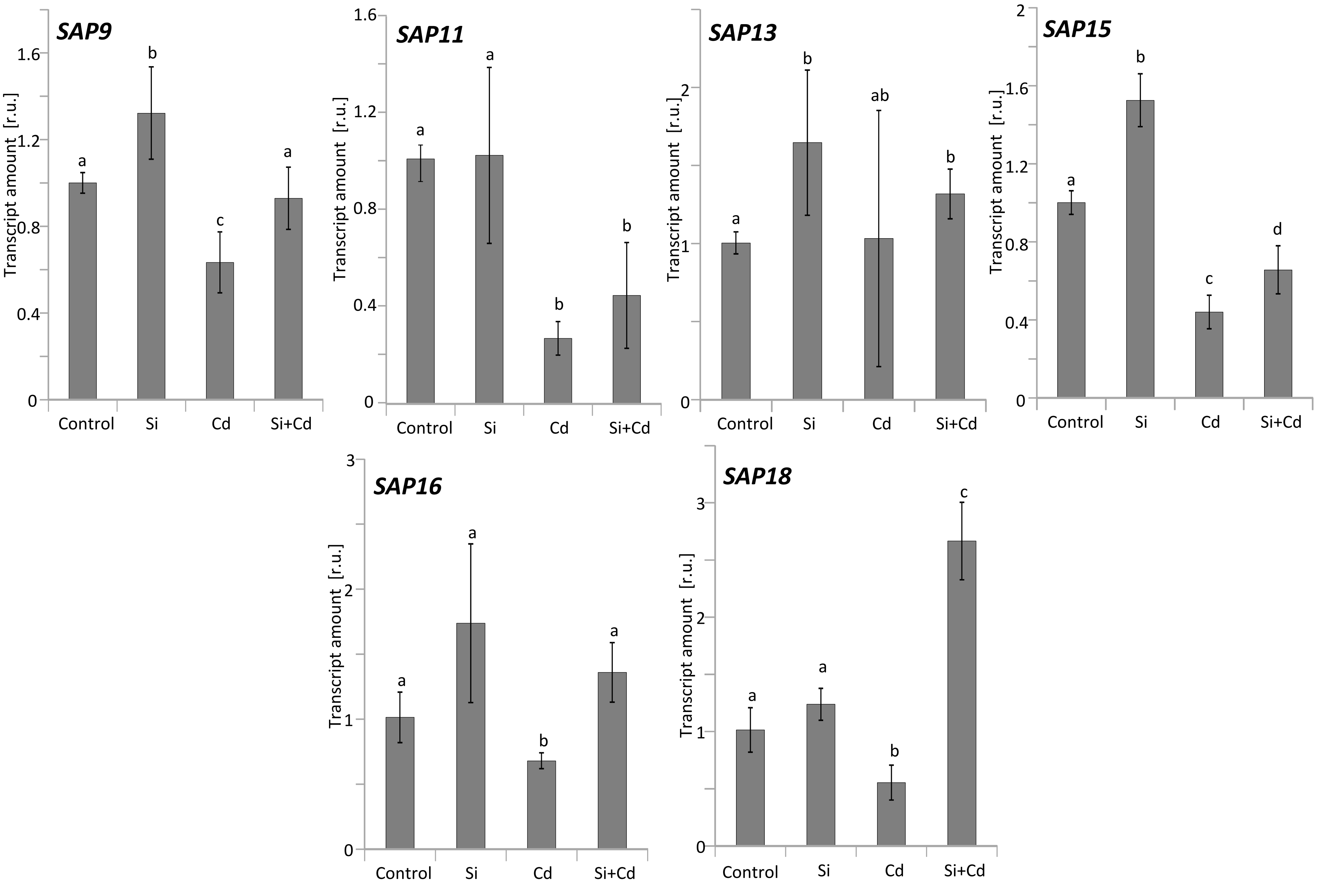

Supplementary Figure S2

Group III: Additive effect (positive or negative) of Si and Cd

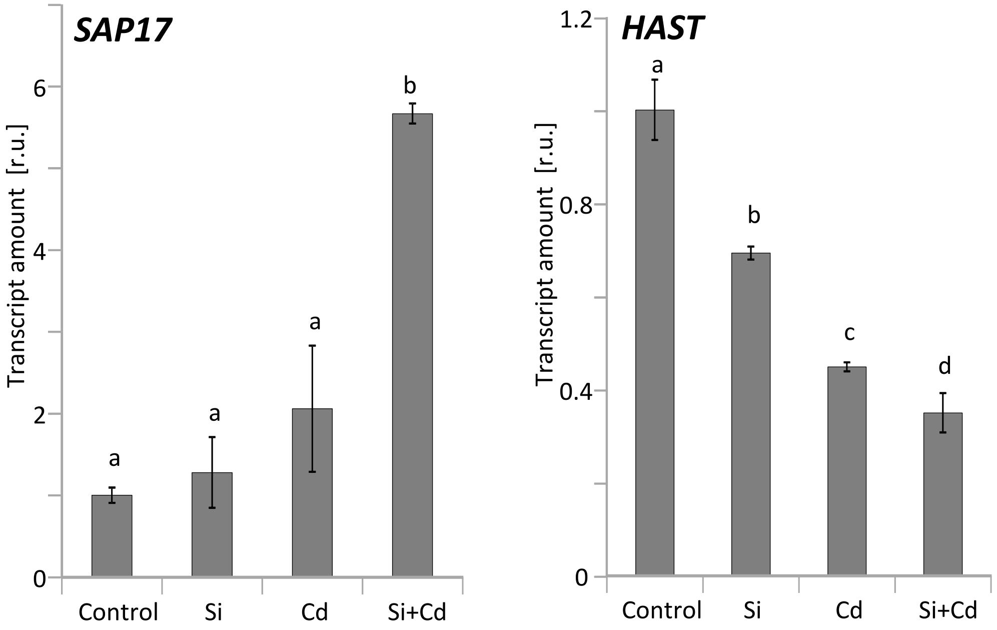

Supplementary Figure S2

Group IV: Complex patterns

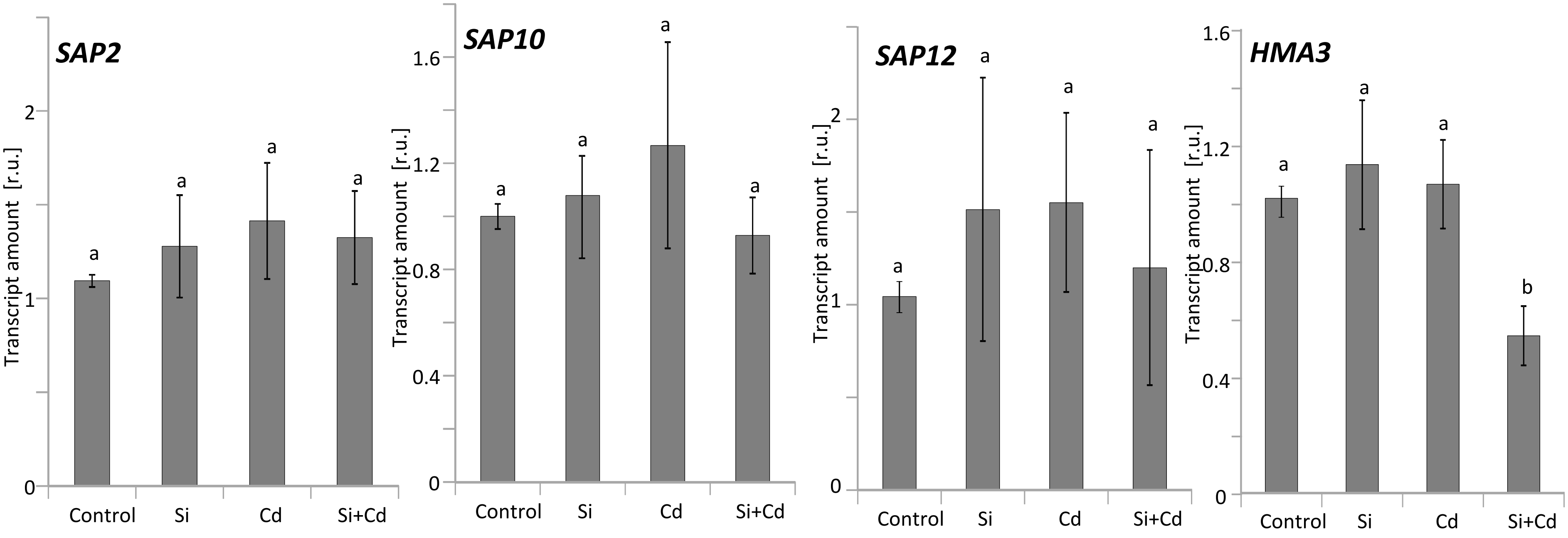

Figure S2: Transcripts abundance in rice roots exposed to Cd toxicity and changes in response 96 hours after Si supply. Transcripts were classified in four response groups according to differential pattern under Cd and/ or Si supply. Group I targets are shown in Fig. 5B. Group II-IV category members are included in Supplementary Figure S2. Figure S2 are means ± S.D. of n=4 from two independent experiments. Letters mark significance groups with $p<0.05$.

Table S1: Sequence of primers used for real-time PCR analysis.

Targets	Accession id	Sequence
OsSAP1	LOC_Os09g31200.1	5′-TTTTAATTGCAAACGGGAGGATA-3′
		5′-TCGATTCTTTTTCCCTCAACCA-3′
OsSAP2	LOC_Os01g52030.1	5′-ACTGCTCGATCTTGCCAACA-3′
		5′-GCAAATCAGCAATCGACCAA-3′
OsSAP3	LOC_Os01g56040.1	5′-GCGCAGTTACGTCTGATTCCT-3′
		5′-CCGGCTCAGGGTTGTTCTT-3′
OsSAP4	LOC_Os02g10200.1	5′-CATCAATAACTGCGGCTTCT-3′
		5′-ACAATGCTATCGATGGAGGA-3′
OsSAP5	LOC_Os02g32840.1	5′-GGCGACAAGCTCAAGGACAAGATC-3′
		5′-CATGGCTGGCTGGCGATTTC-3′
OsSAP6	LOC_Os03g57890.1	5′-AACAACTGTGGCTTCTTTGG-3′
		5′-ACCAAAGGTGTGGAAGACAA-3′
OsSAP7	LOC_Os03g57900.1	5′-CCGGATTTGAGCTCTCCTCTCT-3′
		5′-GCTTGCTACTCAATGTTTGCTTGT-3′
OsSAP8	LOC_Os06g41010.1	5′-CGATGTGGCAACTTGTACTG-3′
		5′-TTAGCCTTGGCAATAGCATC-3′
OsSAP9	LOC_Os07g07350.1	5′-AAACAACTGTGGCTTCTTCG-3′
		5′-CGTAAATGCCTTCTTCTCCA-3′
OsSAP10	LOC_Os07g07400.1	5′-CCGGGGACAGTGACAAAACATC-3′
		5′-CATCACCGTCGTCCCCTCGCCT-3′
OsSAP11	LOC_Os08g39450.1	5′-ACGACTGCAGCTTCGACTACAA-3′
		5′-TGTATTATCCTTTTAGAACCTAACGATCTT-3′
OsSAP12	LOC_Os08g33880.1	5′-CGCCTGCACCTTCGACTT-3′
		5′-CGATCAGCGGGTTCTCCTT-3′
OsSAP13	LOC_Os01g51990.1	5′-TTTATTCACGTGCTCGGTTAGGAT-3′
		5′-TTGGCGCATTTAACCTCATCCGTGA-3′
OsSAP14	LOC_Os03g57920.1	5′-AAGCTGGCCTTCAGGATTTG-3′
		5′-TTGATCGGCTACTTCGATTTGA-3′
OsSAP15	LOC_Os05g23470.1	5′-CAGCACAAATGACAGAACCA-3′
		5′-TTCTTGGCTGAGAATTGAGG-3′
OsSAP16	LOC_Os07g38240.1	5′-GACTGCAACCAGATCGACTT-3′
		5′-TTTGCATTTGGACACTGATG-3′
OsSAP17	LOC_Os09g21710.1	5′-GGAGGCATTTGATCTGTCCAA-3′
		5′-AACCATTTCCCCTGTTAAGAATAGC-3′
OsSAP18	LOC_Os07g07370.1	5′-CACCGGGAACGGCGACAAAGTGGT-3′
		5′-CAAGGTCGTCGCCGACCTCTCGG-3′
OsPCS1	LOC_Os05g34290.1	5′-ACACGATTGATGAGGCAACT-3′
		5′-TTGGGAGGAAGATGATTCAA-3′
OsNAC6	LOC_Os03g60800.1	5′-GGGGTCAAGACTGATTGGAT-3′
		5′-TTGTTGTACAGCCGACACAG-3′
OsNramp1	LOC_Os07g15460.1	5′-CCATGGGAGTAGATTGTTGC-3′
		5′-CGCATCCAAGAAAACTCACT-3′
OsNramp5	LOC_Os07g15370.1	5′-TCCTGGATTCATGGTGTCTT-3′
		5′-CCAATCAGAATCACCCAGAG-3′
OsHAST	LOC_Os03g09970.1	5′-AAGCTCATCGACGAGTTCAC-3′
		5′-CAGGCTGATCAACACCTTCT-3′
OsHMA3	LOC_Os07g12900.1	5′-CTTCATCAAGGGTGGAGATG-3′
		5′-GAACGAGTCGATGCTGAACT-3′
OsHsf31	LOC_Os02g32590.1	5'-CTGATTGCCTCGGATCATTT-3'
		5'-CGTCATCATCTGCATCCATC-3'
OsbHLH	LOC_Os01g06640.1	5'-AGTGGTGGACCAAGTGAAGG-3'
		5'-GCATTCGATGGAGACTTCGT-3'
OsAP2/Erf	LOC_Os07g22730.1	5'-AGCACAAGCAGATCGTCCTC-3'
		5'-ATGGCTAGTTTGCTCGGACA-3'

OsαTubulin	LOC_Os03g51600.1	5′-TCTGAACGTGGATGTCAATG-3′
		5′-GAGATCACTGGAGCGTAGGA-3′
OsActin	LOC_Os03g50885.1	5′-AGGCCGTCCTCTCTCTGTAT-3′
		5′-GGGGAGAGCATATCCTTCAT-3′
